# Exploring factors influencing user perspective of ChatGPT as a technology that assists in healthcare decision making: A cross sectional survey study

**DOI:** 10.1371/journal.pone.0296151

**Published:** 2024-03-08

**Authors:** Avishek Choudhury, Safa Elkefi, Achraf Tounsi

**Affiliations:** 1 Industrial and Management Systems Engineering, Benjamin M. Statler College of Engineering and Mineral Resources, West Virginia University, Morgantown, West Virginia, United States of America; 2 Columbia University School of Nursing, Columbia University Irving Medical Center, New York, New York, United States of America; 3 ADP, New Jersey, United States of America; Federal University of Paraiba, BRAZIL

## Abstract

As ChatGPT emerges as a potential ally in healthcare decision-making, it is imperative to investigate how users leverage and perceive it. The repurposing of technology is innovative but brings risks, especially since AI’s effectiveness depends on the data it’s fed. In healthcare, ChatGPT might provide sound advice based on current medical knowledge, which could turn into misinformation if its data sources later include erroneous information. Our study assesses user perceptions of ChatGPT, particularly of those who used ChatGPT for healthcare-related queries. By examining factors such as competence, reliability, transparency, trustworthiness, security, and persuasiveness of ChatGPT, the research aimed to understand how users rely on ChatGPT for health-related decision-making. A web-based survey was distributed to U.S. adults using ChatGPT at least once a month. Bayesian Linear Regression was used to understand how much ChatGPT aids in informed decision-making. This analysis was conducted on subsets of respondents, both those who used ChatGPT for healthcare decisions and those who did not. Qualitative data from open-ended questions were analyzed using content analysis, with thematic coding to extract public opinions on urban environmental policies. Six hundred and seven individuals responded to the survey. Respondents were distributed across 306 US cities of which 20 participants were from rural cities. Of all the respondents, 44 used ChatGPT for health-related queries and decision-making. In the healthcare context, the most effective model highlights ’Competent + Trustworthy + ChatGPT for healthcare queries’, underscoring the critical importance of perceived competence and trustworthiness specifically in the realm of healthcare applications of ChatGPT. On the other hand, the non-healthcare context reveals a broader spectrum of influential factors in its best model, which includes ’Trustworthy + Secure + Benefits outweigh risks + Satisfaction + Willing to take decisions + Intent to use + Persuasive’. In conclusion our study findings suggest a clear demarcation in user expectations and requirements from AI systems based on the context of their use. We advocate for a balanced approach where technological advancement and user readiness are harmonized.

## Introduction

In the discourse on the emergence and integration of artificial intelligence (AI) in daily life, the rise of generative pre-trained transformers (GPT) like ChatGPT stands as a hallmark of innovation. As an AI model developed by OpenAI, ChatGPT has garnered widespread attention and adoption for its ability to generate human-like text, engaging in conversations and answering queries with a semblance of understanding previously reserved for human intellect.

The application of ChatGPT extends well beyond its initial conception, echoing a common narrative in the evolution of technology where tools are repurposed in manners unforeseen by their developers. ChatGPT, while intended for conversational assistance, has been appropriated for diverse purposes, from drafting legal and academic documents to creating artistic compositions [1–5]. The recontextualization of technology, while innovative, surfaces inherent risks. As with any tool, the efficacy of AI is contingent upon the parameters of its operation—parameters that are defined by data [6]. Such dependencies on data inputs introduces a temporal dimension to its reliability. The AI’s performance today may not be indicative of its performance in the future. The temporal variability is crucial when considering ChatGPT’s role in domains where accuracy is vital, such as healthcare. Just as a treatment’s efficacy may change over time with new medical discoveries, ChatGPT’s responses are subject to the ebb and flow of the data it consumes. A user may receive sound medical advice one month, only to be misinformed the next, should the AI’s data sources become tainted with erroneous information.

User trust in technology is often built over time through consistent, transparent, and reliable performance [7–9]. However, in the context of AI, this trust may become a liability if users become complacent, overlooking the potential for AI responses to degrade as the data landscape shifts. User trust in AI and the extent to which they perceive it to be a helpful decision-making assistant depends on multiple factors such as socio-ethical considerations, technical and design features, user characteristics, and expertise [10, 11]. When users are well-versed in the mechanics of ChatGPT and the principles guiding its responses, they can navigate its capabilities with discernment, appropriately integrating it into their decision-making processes. Conversely, misunderstanding of ChatGPT’s functioning can result in hyped expectations and distorted perception of the technology, leading to unwanted consequences when leveraged for critical applications like healthcare. In other words, if the user is not qualified to validate ChatGPT’s response, the risk or probability of decision errors increases substantially. ChatGPT’s ability to deliver information persuasively also determines how and to what extent people use it. While a convincing articulation can enhance user confidence in the AI’s suggestions, it must be carefully calibrated with the accuracy of the content provided. Persuasiveness without the foundation of reliable and accurate information can lead to misplaced trust and potential misjudgments, especially in high-stakes scenarios such as healthcare decision-making.

The convergence of reliable performance and security protocols also consolidates user trust. On December 1, 2023, Google noted a critical flaw in ChatGPT highlighting the possibility of breaching its training data [12]. GPT models, including ChatGPT, tend to memorize training data, which can lead to privacy concerns [13]. This is particularly concerning if AI models are trained with personal information, as it could lead to the exposure of sensitive data. While efforts are made to align AI models like ChatGPT to prevent the release of large amounts of training data, there is still a risk of data breaches through targeted attacks. As ChatGPT emerges as a potential ally in healthcare decision-making, it is imperative to investigate how users leverage and perceive it.

Our study assesses user perceptions of ChatGPT, particularly of those who used ChatGPT for healthcare-related queries. By examining factors such as competence, reliability, transparency, trustworthiness, security, and persuasiveness of ChatGPT, the research aimed to understand how users rely on ChatGPT for health-related decision-making. Our objective is particularly important in the broader context of AI integration into healthcare systems. As AI technologies like ChatGPT become more prevalent, understanding user perspectives on their effectiveness, trustworthiness, and security becomes crucial.

## Methods

### Ethics statement

The study, bearing the Institutional Review Board (IRB) protocol number 2302725983 and classified as a flex protocol type, received approval from West Virginia University. No identifiers were collected during the study. In compliance with ethical research practices, informed consent was obtained from all participants before initiating the survey. Attached to the survey was a comprehensive cover letter outlining the purpose of the study, the procedure involved, the approximate time to complete the survey, and assurances of anonymity and confidentiality. It also emphasized that participation was completely voluntary, and participants could withdraw at any time without any consequences. The cover letter included contact information of the researchers for any questions or concerns the participants might have regarding the study. Participants were asked to read through this information carefully and were instructed to proceed with the survey only if they understood and agreed to the terms described, effectively providing their consent to participate in the study.

### Data collection

We distributed a web-based semi structured survey to adults in the United States who actively use ChatGPT at least once a month. We collected the data from February 1^st^, 2023, through March 30^th^, 2023. We conducted a soft launch of the survey and collected 40 responses. A soft launch is a small-scale test of a survey before it is distributed to a larger audience. This soft launch aimed to identify any potential issues with the survey, such as unclear or confusing questions, technical glitches, or other problems that may affect the quality of the data collected. The survey was then distributed to a larger audience across the US.

### Instrument

The survey was designed on Qualtrics and was distributed by Centiment, a paid audience-paneling service to reach a broader population [14]. The survey consisted of 17 Likert scale questions (1 = Strongly disagree, 2 = somewhat disagree, 3 = somewhat agree, and 4 = strongly agree) adapted from validated unified theory of acceptance and use of technology (UTAUT) model [15]. The survey also contained two open ended questions as reported in Table 1. To ensure response quality, we included a checking question “We would like to ensure you are reading each question and responding thoughtfully. Please select "Green" as your answer.”

**Table 1:** Survey questions.

Questions	Variable name
How frequently do you use ChatGPT	Use frequency
What is the main reason you use ChatGPT?	Use purpose
**To what extent do you agree or disagree with the following**:	
ChatGPT is competent in providing the information and guidance.	Competent
ChatGPT is reliable in providing consistent and dependable information.	Reliable
ChatGPT is transparent.	Transparent
ChatGPT is trustworthy.	Trustworthy
ChatGPT will not manipulate its responses.	Not manipulate
ChatGPT is secure and protects my privacy and confidential information	Secure
I am willing to use ChatGPT for healthcare-related queries.	ChatGPT for healthcare queries
Benefits of using ChatGPT outweigh any potential risks.	Benefits outweigh risks
ChatGPT helps me make informed and timely decisions.	Help make decisions
I am willing to take decisions based on the recommendations provided by ChatGPT.	Willing to take decisions
I am satisfied with ChatGPT.	Satisfaction
I am willing to use ChatGPT in the future.	Intent to use
ChatGPT can replace human-to-human interaction.	Replace human interaction
ChatGPT is persuasive.	Persuasive
What is the highest degree or level of education you have completed?	Education
*Describe in your own words, the improvements you would like to see in ChatGPT.	Not applicable
*Highlight your concerns with ChatGPT.	Not applicable

*Open ended questions

### Statistical analyses

The analysis was divided into quantitative and qualitative.

The quantitative analyses involved all the Likert scale variables. We first calculated the data’s descriptive statistics and reported the data’s central tendency, dispersion, and inference. We then focused on the dataset corresponding to those who used ChatGPT for making healthcare-related decisions. We calculated the multivariate and pairwise normality of the subset of the data. Given the data distribution and normality violation, we conducted the Bayesian correlation test [16, 17]. In the Bayesian Pearson correlation analyses, the strength and direction of the relationships between various perceived attributes of ChatGPT are quantified using Pearson’s correlation coefficient (r) quantifying the evidence for a correlation against the null hypothesis of no correlation. The Bayesian Linear Regression with Jeffreys-Zellner-Siow (JZS) priors for coefficients was conducted to ascertain the extent to which respondents agree that ChatGPT helps them make informed and timely decisions, denoted as "Help make decisions" [18]. The JZS prior is a default prior representing a compromise between informativeness and non-informativeness, providing a reference analysis less sensitive to the prior choice [18]. The analysis was predicated on a uniform prior distribution, reflecting the absence of a priori preferences or knowledge about the importance of the predictors. This non-informative prior ensures that the posterior distributions are primarily influenced by the data rather than by subjective prior beliefs [18, 19]. Model comparison was achieved by contrasting each model with the best-performing model as a reference. The Bayes Factor (BF_10_) was used to quantify the evidence for each model against the best model, and the coefficient of determination (R^2^) was calculated to assess the proportion of variance explained by the models. BF_10_ provides a measure of evidence of alternative hypothesis (H_1_) over null hypothesis (H_0_), where H_0_ suggests that there is no effect or no difference between the variables being studied, and H_1_ suggests that there is an effect or a difference [20]. Lastly, the posterior distribution of the regression coefficients was summarized, showing the mean, standard deviation (SD), and 95% credible intervals for each factor.

We repeated the same analysis on a subset of our data corresponding to respondents who did not use ChatGPT for health-related decision-making.

Once the best models for each subset were determined, the beta coefficients derived from the optimally performing Bayesian model were further scrutinized to assess autocorrelation using Just Another Gibbs Sampler (JAGS) [21]. The outputs were based on 6000 Markov Chain Monte Carlo draws. Autocorrelation plots and bivariate scatter plots were generated. These plots provided a visual means to examine the independence of the Markov chain samples. Autocorrelation plots were scrutinized for lagged correlations, with an emphasis on identifying significant autocorrelation at various lags that might indicate a lack of convergence or model misspecification [22]. Similarly, bivariate scatter plots were analyzed to detect any systematic patterns indicative of autocorrelation between pairs of parameters.

The open-ended qualitative data was analyzed using content analysis [23]. The question was designed to gather insights on public opinions regarding urban environmental policies. First the unit of analysis was identified as thematic phrases within each response followed by the development of a coding scheme to categorize the survey responses. The primary coding was conducted by one researcher, with periodic validation checks by a second researcher to maintain coding consistency. All coders engaged in iterative discussions to refine and finalize a set of consensus codes, ensuring that the identified themes accurately captured the essence of the participants’ experiences and perspectives. Inter-coder reliability was assessed using Cohen’s Kappa, yielding a coefficient of 0.75, indicative of substantial agreement [24].

## Result

### Data description and understanding

**Fig 1:**
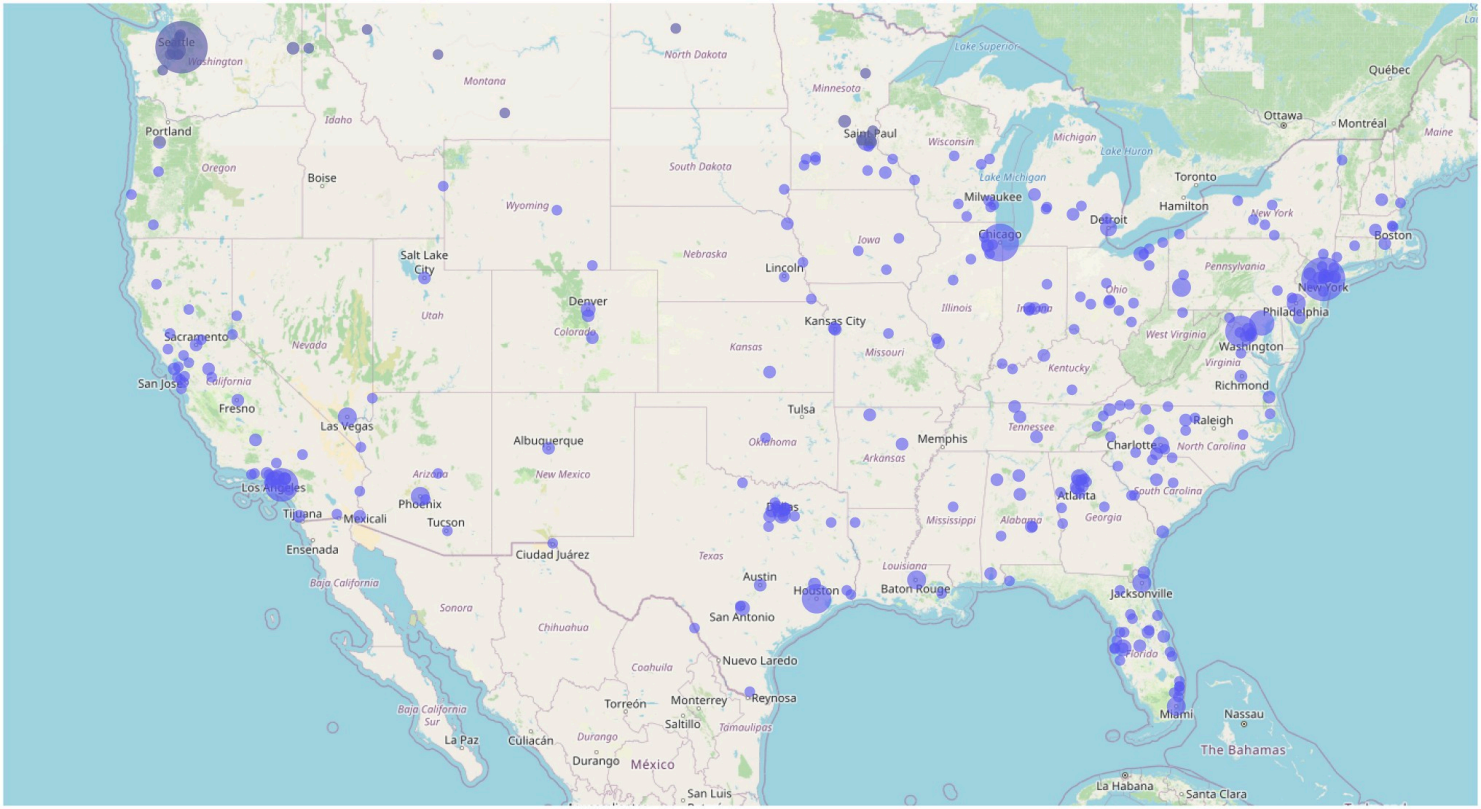
The Figure shows the geographic distribution of study participants from 306 cities across the US. The blue circle size corresponds to the number of responses from each location. The data reveals a high concentration of participants in urban areas across the Eastern Seaboard, parts of the Midwest, and the West Coast, particularly in California, with sparser distribution in the central United States and rural regions.

Six hundred and seven individuals responded to the survey (see S1 Dataset). As illustrated in Fig 1, respondents were distributed across 306 US cities. Only 20 participants were from rural cities. Of all the respondents, 44 used ChatGPT for health-related queries. Other uses of the technology were information gathering (n = 219), entertainment (n = 203), and problem-solving (n = 135), and fun activities (n = 6).

Table 2 shows the descriptive statistics of study variables. The statistics include the mean, standard error of the mean, a 95% confidence interval for the mean (with upper and lower bounds), and standard deviation observed for each variable. Perceptions of ChatGPT’s attributes such as competence, reliability, transparency, trustworthiness, and security were rated positively, with all means exceeding 3.0.

**Table 2:** Descriptive statistics.

	Mean	Std. error of mean	95% confidence interval mean		Std. Deviation
			Upper	Lower	
Competent	3.19	0.03	3.26	3.13	0.82
Reliable	3.16	0.03	3.22	3.09	0.80
Transparent	3.12	0.03	3.19	3.05	0.86
Trustworthy	3.17	0.03	3.23	3.10	0.84
Not manipulate	3.09	0.04	3.17	3.03	0.88
Secure	3.27	0.03	3.34	3.21	0.81
ChatGPT for healthcare queries	3.097	0.035	3.165	3.029	0.855
Benefits outweigh risks	3.204	0.033	3.268	3.140	0.801
Helps make decisions	3.257	0.032	3.320	3.194	0.790
Satisfaction	3.244	0.031	3.305	3.183	0.764
Willing to take decisions	3.130	0.033	3.195	3.065	0.815
Intent to use	3.379	0.031	3.439	3.319	0.757
Replace human interaction	2.292	0.037	3.001	2.857	0.901
Persuasive	2.979	0.033	3.044	2.913	0.819
Education	2.855	0.036	2.925	2.785	0.884

***p<0.001

### Quantitative findings using subset of the data who used ChatGPT for healthcare related queries

#### Correlation analyses

**Fig 2:**
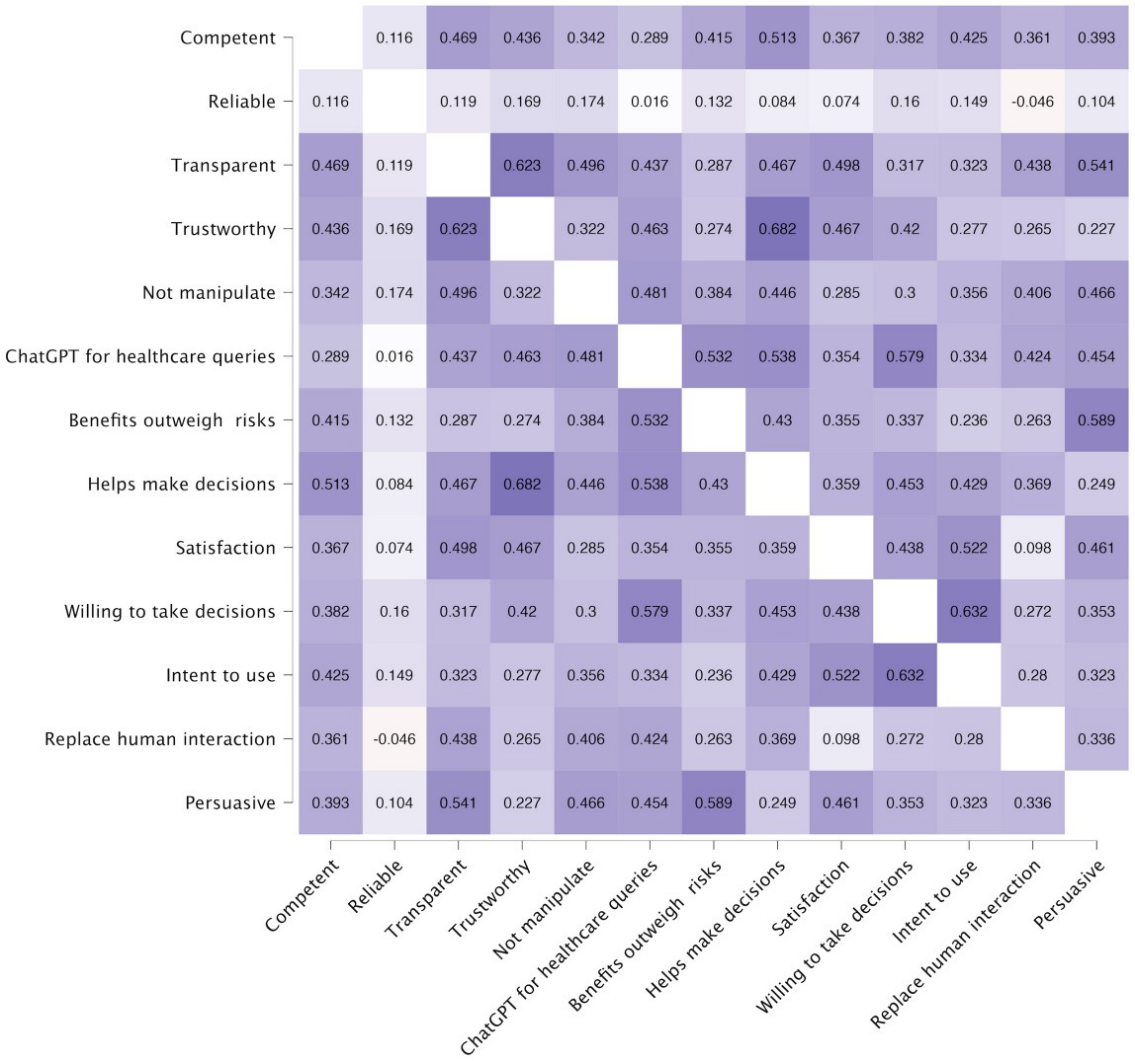
Heatmap of correlation coefficients between user perception factors of ChatGPT, with actual usage for healthcare-related queries.

This sections reports finding based on 44 respondents who used ChatGPT for healthcare-related queries. Fig 2 presents a nuanced landscape of significant correlations among various perceived attributes of ChatGPT as evaluated by users employing the AI for health-related inquiries.

A notable significant correlation exists between ’Transparent’ and ’Trustworthy’, suggesting a robust relationship where the clarity of ChatGPT’s processes and intentions is strongly associated with users’ trust. ’Trustworthy’ also shares a substantial correlation with ’Helps make decisions’, indicating that trust in ChatGPT is crucial for users considering its advice in making health-related decisions.

Additionally, ’Secure’ exhibits very strong evidence of correlation with ’ChatGPT for healthcare queries’, pointing to security as a pivotal factor for users when consulting ChatGPT for health concerns. The significant correlation between ’Secure’ and ’Not manipulate’ also highlights the interdependence of security and the non-manipulative nature of responses in fostering a safe environment for health-related interactions.

’Benefits outweigh risks’ has a very strong correlation with ’ChatGPT for healthcare queries’, which could indicate that users who perceive higher benefits than risks are more likely to engage with ChatGPT for health-related purposes. ’Intent to use’ shows a strong positive correlation with ’Willing to take decisions’, suggesting that users who intend to use ChatGPT are also more likely to trust it for decision-making. The correlation of ’Intent to use’ with ’Satisfaction’ and ’ChatGPT for healthcare queries’ emphasizes the connection between future use intentions, current satisfaction levels, and the perceived utility of ChatGPT in health-related matters. Lastly, ’Persuasive’ (N) demonstrates very strong evidence of correlation with ’ChatGPT for healthcare queries’, indicating that the ability of ChatGPT to influence user opinions or actions is significantly related to its use for health queries.

**Fig 3:**
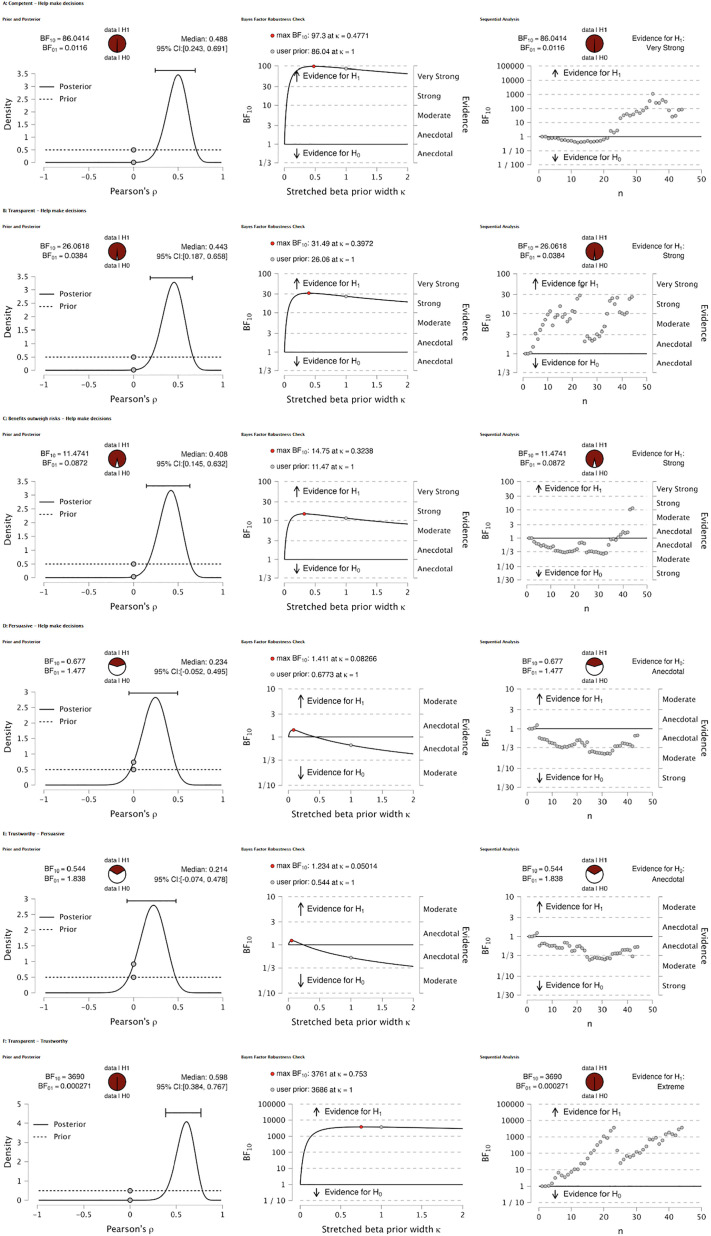
Bayesian correlation sequential analyses of ChatGPT’s attributes and their influence on decision-making assistance. **A**: Correlation between perceived competence of ChatGPT and its assistance in decision-making, indicating very strong evidence for the positive association (BF_10_ = 86.04); **B**: Association between perceived transparency of ChatGPT and its aid in decision-making, demonstrating strong evidence for the correlation (BF_10_ = 26.06); **C**: Relationship between the perceived benefits outweighing risks when using ChatGPT for decision-making, showing strong evidence for the correlation (BF_10_ = 11.47); **D**: Correlation between perceived persuasiveness of ChatGPT and its impact on decision-making, with anecdotal evidence for the association (BF_10_ = 0.67); **E**: Correlation between perceived trustworthiness and persuasiveness of ChatGPT, suggesting anecdotal evidence for their combined influence on decision-making assistance (BF_10_ = 0.54); **F**: Analysis of the relationship between transparency and trustworthiness in ChatGPT, with extreme evidence supporting a very strong correlation (BF_10_ = 3690).

As illustrated in Fig 3, in the subsequent in-depth analysis of selected pairwise correlation, we investigated the relationship between ‘Help make decisions’ with ‘Competence,’ ‘Transparent,’ ‘Benefits outweigh risks,’ and ‘Persuasive’. We also explored pairwise correlation of ‘Trustworthy’ with ‘Transparent’ and ‘Persuasive’. Prior and posterior distributions for Pearson’s correlation coefficient were generated to elucidate the relationship between variables. A robustness check was conducted to ensure the stability of the Bayes Factor across varying priors. This analysis confirmed the strength of the evidence for the alternative hypothesis (H1).

As additional data points were sequentially integrated into the analysis, the evidence for a positive correlation between the variable remained robustly within the threshold for extreme, very strong, and anecdotal evidence. This pattern persisted across the accumulation of data points, indicating that the observed correlation was not a consequence of sample size but a persistent trend within the data.

For the attribute of competence, the analysis indicated very strong evidence (BF_10_ = 86.04) for the hypothesis that ChatGPT’s competence positively influences its ability to help users make decisions. This was further corroborated by the robustness checks, which consistently showed substantial support for this relationship across a spectrum of priors. The transparency of ChatGPT also appeared to be a significant factor, with a Bayes Factor suggesting strong evidence (BF_10_ = 26.06) for its correlation with aiding decision-making. This aligns with the notion that transparency in the functioning of AI systems may bolster user trust and reliance on their decision-making capabilities.

In the case of the perceived benefits outweighing risks, there was strong evidence (BF_10_ = 11.47) supporting the relationship with decision-making assistance. Users who felt that the advantages of using ChatGPT surpassed any potential risks were more likely to consider it helpful in making decisions. However, when examining the attribute of persuasiveness, the evidence was merely anecdotal (BF_10_ = 0.67), indicating a weak relationship with decision-making aid. This suggests that while ChatGPT’s ability to persuade may be noticed by users, it does not significantly influence their reliance on the system for decision-making support.

The trustworthiness attribute, when analyzed in conjunction with persuasiveness, showed a similarly modest level of evidence (BF_10_ = 0.54), again suggesting that these factors alone do not strongly predict ChatGPT’s perceived utility in decision-making. The most compelling result was observed in the correlation between transparency and trustworthiness, where an extreme Bayes Factor (BF_10_ = 369) was noted, indicating an exceptionally strong relationship between these attributes. This underscores the integral role of transparent operations and trust in the perceived effectiveness of AI systems like ChatGPT in supporting user decisions. This conclusion aligns with the overarching narrative of the primary analysis and adds a layer of depth to the understanding of user perceptions of ChatGPT within the specific context of health-related inquiries.

### Bayesian linear regression analysis

Table 3 presents a Bayesian linear regression comparing various models to ascertain the factors influencing decision-making when ChatGPT is not used for healthcare-related queries. P(M) represents the prior probability of each model before data observation, assuming a non-informative or uniform prior. P(M|data) denotes the posterior probability, reflecting the model’s probability after considering the observed data. The Bayes Factor (BF_M_) and Bayes Factor_10_ (BF_10_) provide evidence strength for each model against a baseline model, with BF_M_ referencing the null model and BF_10_ comparing each alternative hypothesis to the null hypothesis. The coefficient of determination (R^2^) indicates the proportion of variance in decision-making that is predictable from the independent variables in each model. The models are ranked by the strength of evidence.

**Table 3:** Comparative Bayesian analysis of decision-making models (n = 44).

Models → Help make decision	P(M)	P(M|data)	BF_M_	Log(BF_10_)	R^2^
Trustworthy	4.76x10^-3^	0.09	21.73	10.40	0.46
Trustworthy + Benefits outweigh risks	7.33 x 10^−4^	0.03	45.47	11.20	0.53
Trustworthy + ChatGPT for healthcare queries	7.33 x 10^−4^	0.03	42.91	11.14	0.53
Trustworthy + Intent to use	7.33 x 10^−4^	0.03	42.25	11.13	0.53
Competent + Trustworthy	7.33 x 10^−4^	0.02	34.23	10.92	0.52
Trustworthy + Not manipulate	7.33 x 10^−4^	0.02	34.09	10.92	0.52
Trustworthy + Replace human interaction	7.33 x 10^−4^	0.01	16.13	10.19	0.50
Competent + Trustworthy + ChatGPT for healthcare queries	7.33 x 10^−4^	0.01	61.26	11.52	0.57
Trustworthy + Benefits outweigh risks + Intent to use	1.83 x 10^−4^	0.01	60.46	11.51	0.57

P(M) = prior probability of the model before observing the data (non-informative)

P(M|data) = posterior probability of the model given the observed data.

BF_M_ = Bayes Factor of the model relative to a baseline (null) model

F_10_ = Bayes Factor_10_ refers to the comparison between the alternative hypothesis (H1) and the null hypothesis (H0)

As shown in the Table the model comprising " Competent + Trustworthy + ChatGPT for healthcare queries" emerged as the most robust, reflected by the highest Bayes Factor in relation to the null model, suggesting substantial evidence in favor of this model compared to the alternative models considered. The coefficient of determination (R^2^) for this model was 0.57, indicating that approximately 57% of the variance in the use of ChatGPT for making decisions was accounted for by the predictors included in this model.

**Fig 4:**
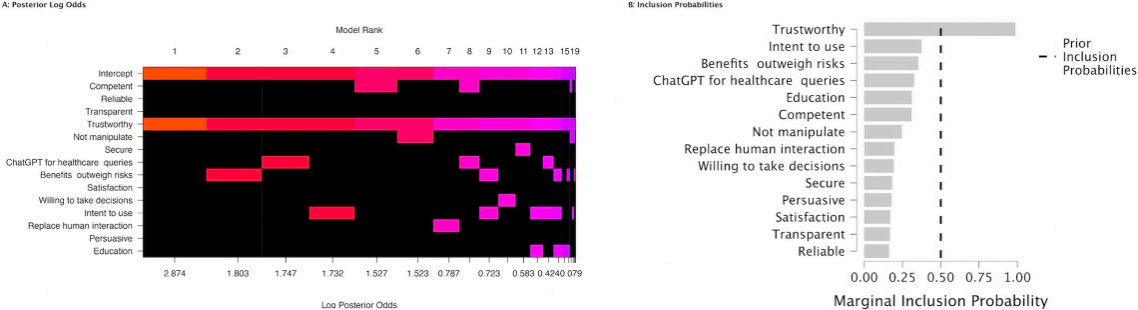
Bayesian model analysis of user perception of ChatGPT. **A**: The left panel displays model ranks based on log posterior odds, highlighting the most influential factors determining the intention to use ChatGPT for healthcare inquiries. The colored squares (non-black spaces) indicate the included covariates for a given model, the null model being purple; **B**: The right panel presents the marginal inclusion probabilities for each factor.

Fig 4 shows the posterior log odds and marginal inclusion probabilities. According to the posterior summaries (Table 4) ‘Trustworthy’ stands out with a posterior inclusion probability of 0.99, which indicates near certainty that trustworthiness is relevant after considering the data. The Log(BF_inclusion_) for ’Trustworthy’ is substantially positive at 4.42, which provides strong evidence for its inclusion in the model over the null hypothesis. Its effect size, represented by the mean coefficient, is 0.50 with a relatively small standard deviation of 0.14, indicating a strong and precise estimate of its impact. The 95% credible interval ranges from 0.22 to 0.78, which does not cross zero and implies that the positive effect of trustworthiness on decision-making is statistically significant with high confidence.

**Table 4:** Posterior analysis of predictors influencing decision-making using Bayesian linear regression (n = 44).

Coefficient	P(incl)	P(excl)	P (incl|data)	P (excl|data)	Log (BF_inclusion_)	Mean	Std. Dev	95% Credible interval	
								Lower	Upper
Intercept	1.00	0.00	1.00	0.00	0.00	3.23	0.09	3.04	3.39
Competent	0.50	0.50	0.31	0.69	-0.80	0.06	0.11	-1.16x10^-3^	0.35
Reliable	0.50	0.50	0.16	0.84	-1.64	-0.01	0.06	-0.23	0.07
Transparent	0.50	0.50	0.17	0.83	-1.60	-0.01	0.06	-0.21	0.09
Trustworthy	0.50	0.50	0.99	0.01	4.42	0.50	0.14	0.22	0.78
Not manipulate	0.50	0.50	0.25	0.75	-1.12	0.03	0.08	-7.09x10^-3^	0.32
Secure	0.50	0.50	0.18	0.82	-1.49	0.01	0.07	-0.11	0.21
ChatGPT for healthcare queries	0.50	0.50	0.33	0.67	-0.72	0.07	0.13	-0.03	0.40
Benefits outweigh risks	0.50	0.50	0.35	0.65	-0.60	0.07	0.12	-0.01	0.35
Satisfaction	0.50	0.50	0.17	0.83	-1.58	-0.01	0.06	-0.21	0.06
Willing to take decisions	0.50	0.50	0.19	0.81	-1.43	0.01	0.07	-0.05	0.25
Intent to use	0.50	0.50	0.37	0.63	-0.51	0.08	0.13	-0.01	0.37
Replace human-to-human interaction	0.50	0.50	0.20	0.80	-1.41	0.02	0.07	-0.08	0.21
Persuasive	0.50	0.50	0.18	0.82	-1.53	-0.02	0.08	-0.30	0.05
Education	0.50	0.50	0.31	0.69	-0.79	-0.05	0.09	-0.27	0.00

**Fig 5:**
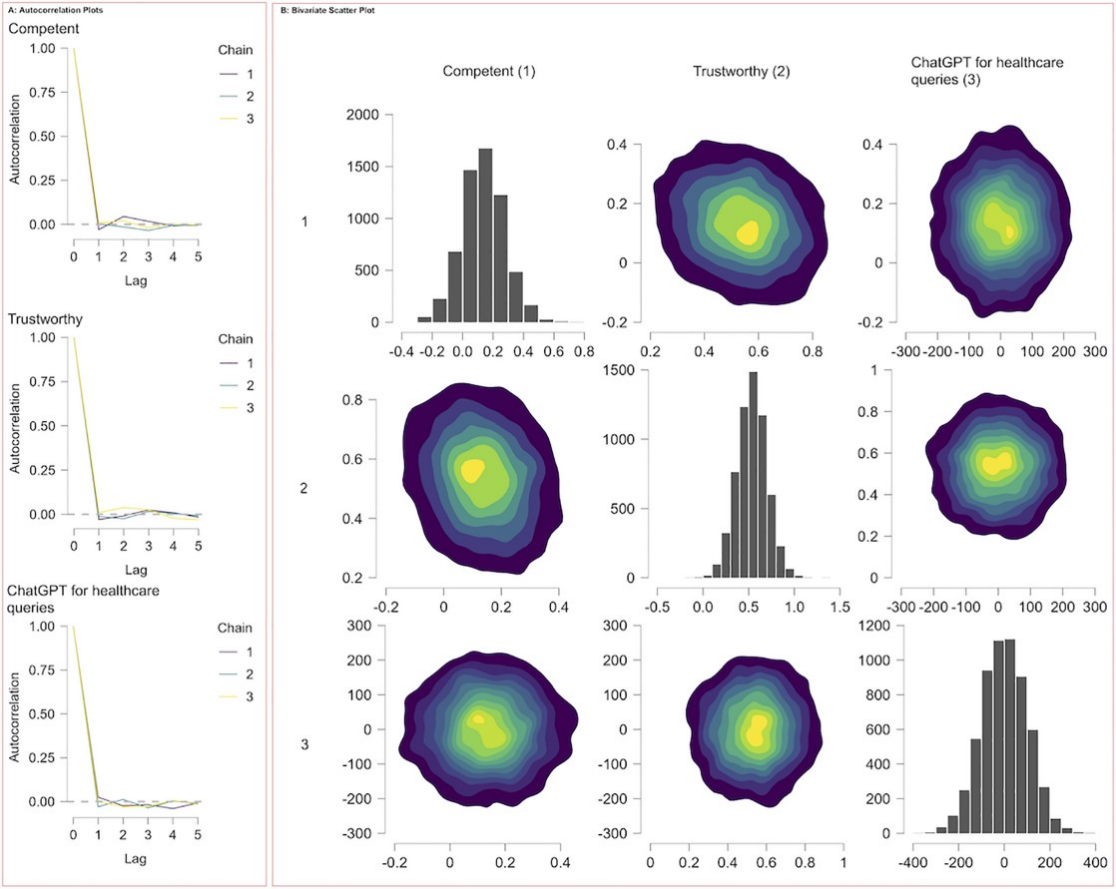
MCMC output analysis from JAGS. **Panel A**: displays autocorrelation plots for three components, showing low correlation across lags for multiple chains, indicative of effective sampling. **Panel B**: presents bivariate scatter plots and marginal histograms for parameter pairs, reflecting their joint and marginal distributions, essential for understanding parameter interactions and individual uncertainties within the model.

Fig 5 presents the diagnostics from a Markov Chain Monte Carlo simulation executed via JAGS, focusing on the autocorrelation within the model’s parameters. The autocorrelation plots in Panel A display a rapid decline to near-zero levels by lag 1 across multiple chains for several metrics, an indication that the chains are well-mixed and that the samples are independent of each other. This low autocorrelation persists across further lags, which is indicative of an efficient exploration of the parameter space and suggests robust sampling performance. The bivariate scatter plots and accompanying histograms in Panel B reinforce this interpretation, showing tight, bell-shaped distributions and clear peaks within the contour plots, characteristics that are commonly associated with effective sampling and convergence within Markov Chain Monte Carlo simulations. Collectively, these diagnostic plots suggest that the model exhibits low autocorrelation, implying reliable and accurate parameter estimation.

### Quantitative findings using subset of the data who did not use ChatGPT for healthcare related queries

#### Bayesian correlation analysis

**Fig 6:**
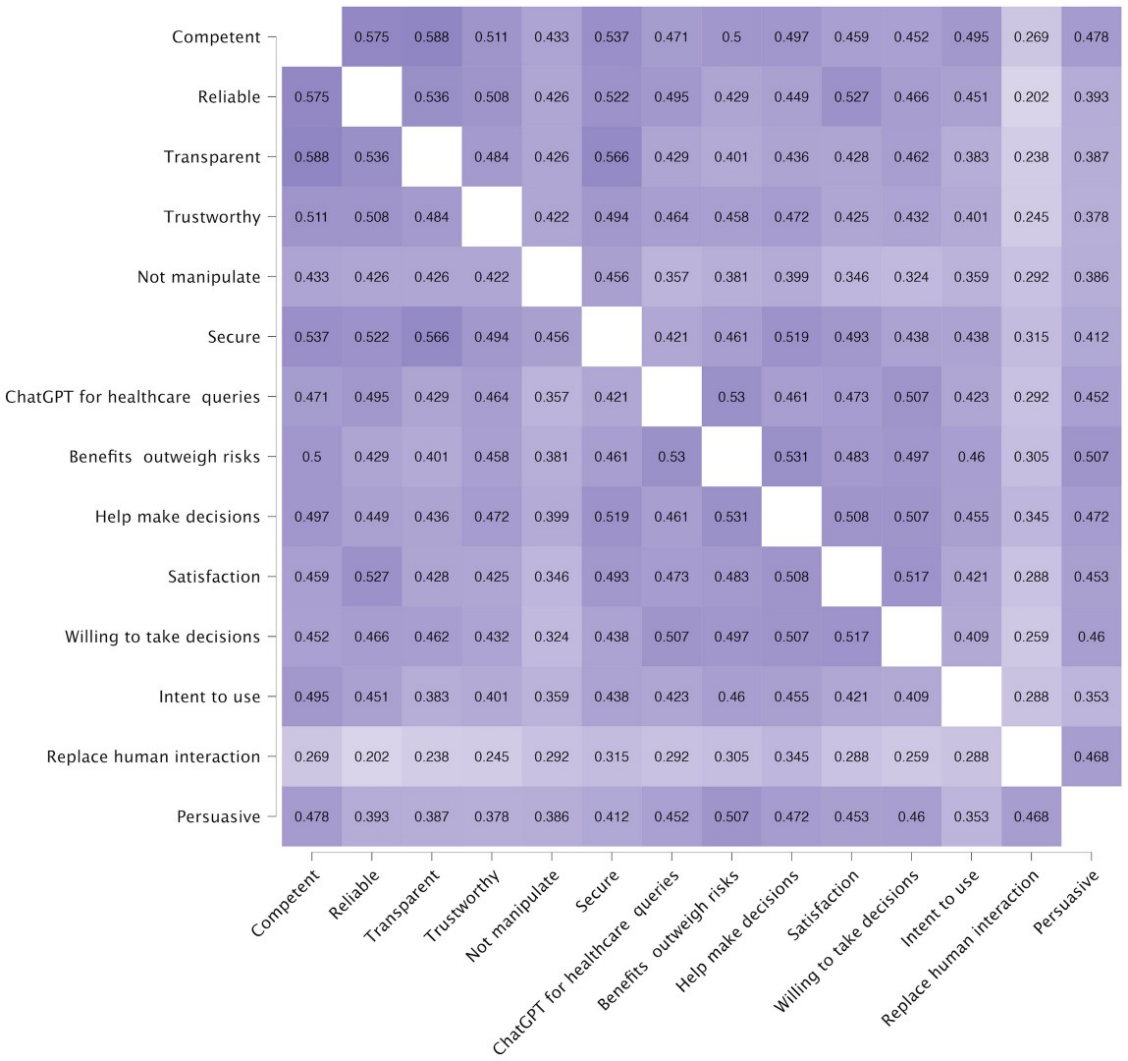
Heatmap of correlation coefficients between user perception factors of ChatGPT, without actual usage for healthcare-related queries.

This sections reports finding based on 563 respondents who did not use ChatGPT for healthcare-related queries. The correlation analysis (Fig 6) indicates that factors related to the perception of ChatGPT as competent, reliable, and trustworthy are moderately to highly correlated with the intent to use it for healthcare-related decisions. Notably, ’Trustworthy’ is a central factor, exhibiting moderate correlations with ’Secure’, ’Benefits outweigh risks’, and ’Help make decisions’, which underscores its importance in influencing user acceptance. ’Help make decisions’ is notably correlated with ’Satisfaction’ and ’Willing to take decisions’, suggesting that the perceived effectiveness of ChatGPT in aiding decision-making significantly relates to user satisfaction and their willingness to engage with the system. Lower correlations with ’Replace human interaction’ across the board suggest this is a less central concern to users relative to other factors.

#### Bayesian linear regression analysis

According to Table 5, the model featuring ’Trustworthy + Secure + Benefits outweigh risks + Satisfaction + Willing to take decisions + Intent to use + Persuasive’ has the highest Bayes Factor of 1522.67, and its Log Bayes Factor is 157.27, with a posterior probability of 0.03. The Bayes Factor indicates very strong evidence in favor of this model compared to the null model, suggesting that the combination of these predictors has a compelling influence on the decision-making process. This model does not include ’Competent’ or ’Not manipulate’, which suggests that while competence and non-manipulative nature may be important, they are not as critical as the other factors in this context. The absence of ’Replace human interaction’ in the top model also suggests that this factor is not a primary concern for users when it comes to using ChatGPT for healthcare decisions.

**Table 5:** Comparative Bayesian analysis of decision-making models in non-healthcare contexts (n = 563).

Models → Help make decision	P(M)	P(M|data)	BF_M_	Log (BF_10_)	R^2^
Trustworthy + Secure + Benefits outweigh risks + Satisfaction + Willing to take decisions + Intent to use + + Replace human interaction + Persuasive	2.22 x 10^−5^	0.03	1438.97	157.21	0.47
Trustworthy + Secure + Benefits outweigh risks + Satisfaction + Willing to take decisions + Intent to use + Persuasive	1.94 x 10^−5^	0.03	1522.67	157.27	0.47
Competent + Trustworthy + Secure + Benefits outweigh risks + Satisfaction + Willing to take decisions + Intent to use + Replace human interaction + Persuasive	3.33 x 10^−5^	0.02	763.77	156.59	0.47
Competent + Trustworthy + Secure + Benefits outweigh risks + Satisfaction + Willing to take decisions + Intent to use + Replace human interaction	2.22 x 10^−5^	0.02	1086.39	156.94	0.47
Trustworthy + Secure + Benefits outweigh risks + Satisfaction + Willing to take decisions + Intent to use + Replace human interaction	1.94 x 10^−5^	0.02	1092.32	156.95	0.47
Competent + Trustworthy + Secure + Benefits outweigh risks + Satisfaction + Willing to take decisions + Replace human interaction	1.94 x 10^−5^	0.02	811.28	156.66	0.47
Trustworthy + Not manipulate + Secure + Benefits outweigh risks + Satisfaction + Willing to take decisions + Intent to use + Replace human interaction + Persuasive	3.33 x 10^−5^	0.01	441.16	156.05	0.47
Competent + Trustworthy + Secure + Benefits outweigh risks + Satisfaction + Willing to take decisions + Intent to use + Persuasive	2.22 x 10^−5^	0.01	645.24	156.43	0.47
Competent + Trustworthy + Not manipulate + Secure + Benefits outweigh risks + Satisfaction + Willing to take decisions + Intent to use + Replace human interaction + Persuasive	6.66 x 10^−5^	0.01	199.75	155.26	0.48

P(M) = prior probability of the model before observing the data (non-informative)

P(M|data) = posterior probability of the model given the observed data.

BF_M_ = Bayes Factor of the model relative to a baseline (null) model

BF_10_ = Bayes Factor_10_ refers to the comparison between the alternative hypothesis (H1) and the null hypothesis (H0)

**Fig 7:**
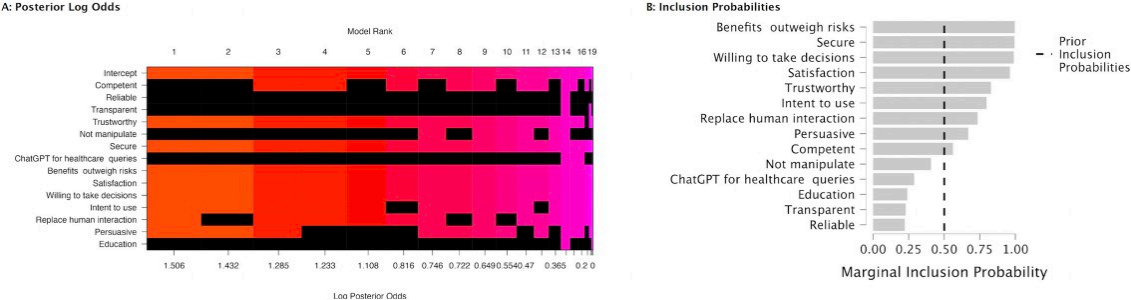
Bayesian model analysis of non-healthcare user perception of ChatGPT. **A**: The left panel displays model ranks based on log posterior odds, highlighting the most influential factors determining the intention to use ChatGPT for healthcare inquiries. The colored squares (non-black spaces) indicate the included covariates for a given model, the null model being purple; **B**: The right panel presents the marginal inclusion probabilities for each factor.

Fig 7 shows the posterior log odds and marginal inclusion probabilities. Table 6 summarizes the posterior distributions of regression coefficients for each variable contributing to decision-making efficacy. The posterior summaries of coefficients indicates that ’Secure’, ’Benefits outweigh risks’, ’Satisfaction’, and ’Willing to take decisions’ are the most influential predictors of decision-making in this context.

**Table 6:** Posterior analysis of predictors influencing decision-making using Bayesian linear regression (n = 563).

Coefficient	P(incl)	P(excl)	P (incl|data)	P (excl|data)	Log (BF_inclusion_)	Mean	Std. Dev	95% Credible interval	
								Lower	Upper
Intercept	1.00	0.00	1.00	0.00	0.00	3.26	0.02	3.22	3.31
Competent	0.50	0.50	0.56	0.44	0.25	0.04	0.05	0.00	0.14
Reliable	0.50	0.50	0.22	0.78	-1.25	1.88x10^-3^	0.02	-0.04	0.06
Transparent	0.50	0.50	0.23	0.77	-1.21	3.30x10^-3^	0.02	-0.03	0.07
Trustworthy	0.50	0.50	0.83	0.17	1.57	0.08	0.05	0.00	0.15
Not manipulate	0.50	0.50	0.41	0.59	-0.38	0.02	0.03	-1.92x10^-5^	0.10
Secure	0.50	0.50	0.99	6.71x10^-3^	5.00	0.15	0.04	0.08	0.24
ChatGPT for healthcare queries	0.50	0.50	0.29	0.71	-0.91	0.01	0.03	-0.01	0.09
Benefits outweigh risks	0.50	0.50	1.00	4.03x10^-3^	5.51	0.16	0.04	0.08	0.24
Satisfaction	0.50	0.50	0.96	0.04	3.21	0.13	0.05	0.00	0.20
Willing to take decisions	0.50	0.50	0.99	0.01	4.48	0.14	0.04	0.06	0.22
Intent to use	0.50	0.50	0.80	0.20	1.37	0.08	0.05	0.00	0.16
Replace human-to-human interaction	0.50	0.50	0.73	0.27	1.02	0.05	0.04	-7.64x10^-4^	0.12
Persuasive	0.50	0.50	0.67	0.33	0.70	0.06	0.05	-1.05x10^-3^	0.15
Education	0.50	0.50	0.24	0.76	-1.15	4.11x10^-3^	0.02	-0.02	0.05

**Fig 8:**
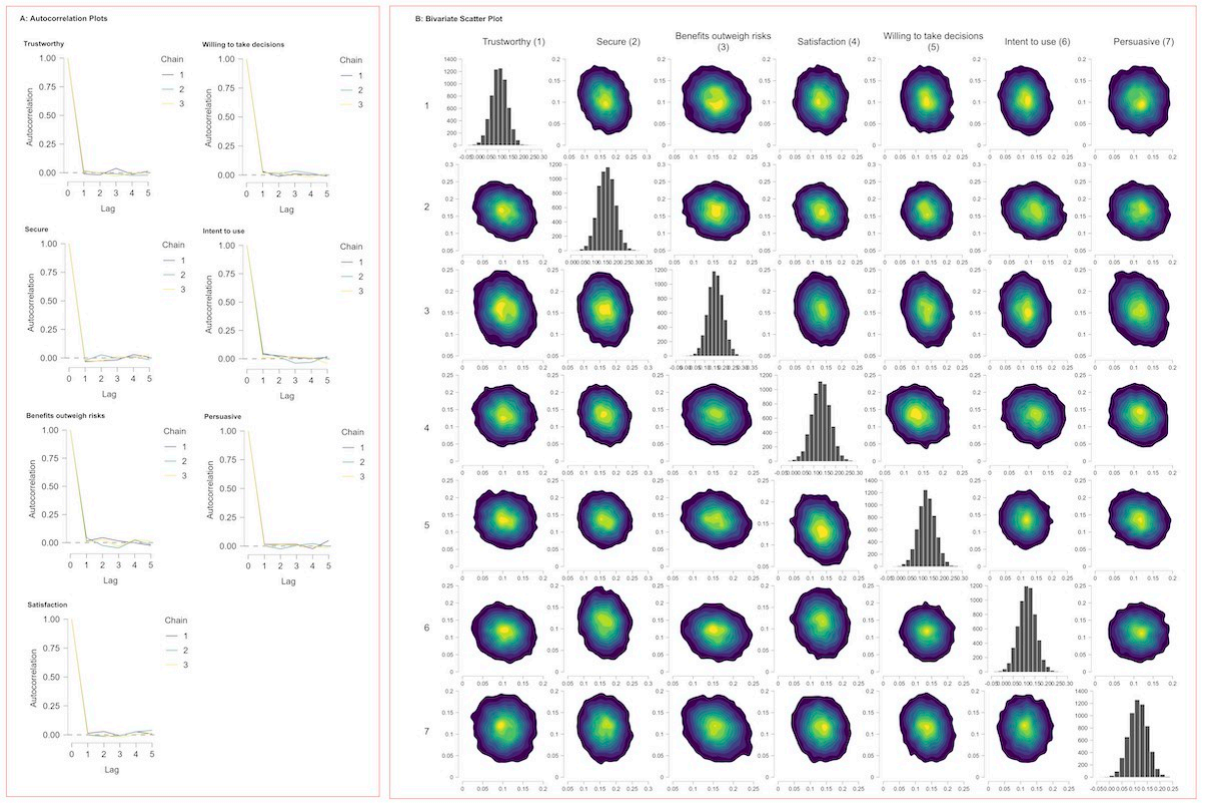
MCMC output analysis from JAGS. **Panel A**: shows autocorrelation plots for seven metrics, indicating chain independence with low autocorrelation at increased lags. **Panel B**: presents bivariate scatter plots and marginal histograms for parameter pairs, reflecting their joint and marginal distributions, essential for understanding parameter interactions and individual uncertainties within the model.

Fig 8 imply that the MCMC simulation is free from significant autocorrelation, thereby confirming the dependability of the statistical estimates produced. The autocorrelation graphs in panel A shows a swift drop-off to negligible levels after the initial lag in all chains, which is a strong indicator of chain sample independence. The bivariate scatter plots in panel B exhibit dense central clustering with well-defined peaks, and the marginal histograms show symmetric distributions, both pointing towards a well-converged MCMC process.

#### Qualitative findings

Distinct patterns emerged when contrasting the concerns of users employing the ChatGPT for healthcare-related inquiries against those using it for other purposes. Both cohorts expressed concerns over privacy, a testament to the overarching necessity for robust data security protocols in AI interactions. However, the nuances of their feedback reveal divergent emphases reflective of the context of use.

Users engaging with ChatGPT for healthcare-related matters demonstrate a pronounced emphasis on safety and trust. This is indicative of the critical nature of healthcare information and the consequential outcomes dependent on its reliability. Concerns such as the AI becoming overly intelligent suggest apprehensions about the delegation of health-related decision-making to artificial entities, raising questions about the ethical bounds of AI in sensitive sectors. Moreover, while both sets of users highlight the importance of content quality, the healthcare user group uniquely underlines the dual dimensions of quantity and quality. This emphasis may underscore the necessity for comprehensive yet reliable health information that AI platforms like ChatGPT are expected to deliver. In contrast, the feedback from users not engaged in healthcare queries with ChatGPT tends to span a broader spectrum of technical and usability enhancements. Calls for a more user-friendly interface, and improved load times. Users also express the need for more human-like interactions, suggesting an aspiration for AI to bridge the gap between technological functionality and human relationality. Notably, users from the non-healthcare cohort voiced concerns about potential political or ideological biases in AI responses. This highlights a distinct concern for objectivity and neutrality, which, while important in all contexts, appears particularly salient for users seeking general information and assistance from ChatGPT.

The comparative analysis reveals that while the foundation of user trust in AI is built on privacy and accuracy, the specific context of use—healthcare versus general inquiries—exerts a significant influence on the nuances of user concerns. For healthcare-related AI applications, the implications are clear: there is a critical need for heightened measures of accuracy, security, and ethical considerations, aligning with the sensitive nature of healthcare information and decision-making processes.

## Discussion

### Main findings

In the realm of healthcare decision-making, the prominence of trustworthiness, as indicated by our study, is particularly instructive. In the healthcare context (Tables 3 and 4), the most effective model highlights ’Competent + Trustworthy + ChatGPT for healthcare queries’, underscoring the critical importance of perceived competence and trustworthiness specifically in the realm of healthcare applications of ChatGPT. On the other hand, the non-healthcare context (Tables 5 and 6) reveals a broader spectrum of influential factors in its best model, which includes ’Trustworthy + Secure + Benefits outweigh risks + Satisfaction + Willing to take decisions + Intent to use + Persuasive’. While trustworthiness forms a common thread in both contexts, healthcare decision-making prioritizes the specific applicability and competence of ChatGPT in healthcare, whereas in non-healthcare settings, a wider range of factors influences the adoption of technology, reflecting a more varied and holistic approach to decision-making.

### Perception of users using ChatGPT for healthcare queries

The heightened importance of perceived competence and user trust in ChatGPT for those utilizing it for healthcare queries, as indicated in our study, can be understood through the lens of the critical nature of healthcare decisions and the evolving role of AI in such sensitive contexts. In healthcare, where decisions carry significant consequences, the perceived competence and trustworthiness of AI tools like ChatGPT are paramount for users. The emphasis on competence stems from the necessity for accurate, relevant, and reliable information, given the potential adverse outcomes of incorrect advice in health-related matters. Trust forms the foundation of user reliance on AI in healthcare, as it assures users of the tool’s capability to handle sensitive information responsibly and ethically. The integration and acceptance of ChatGPT in healthcare thus hinge not just on its technological sophistication but critically on its ability to be seen as a competent and trustworthy partner in healthcare decision-making. This focus is particularly acute in healthcare due to the high stakes involved, contrasting with other contexts where the implications of misinformation may be less severe. Consequently, ensuring that ChatGPT is perceived as both knowledgeable and reliable is essential for its successful adoption in healthcare settings.

The perception of ChatGPT as competent and trustworthy in healthcare brings with it a complex interplay of benefits and risks. On the one hand, it promises improved access to healthcare information, especially valuable in remote or underserved areas, and acts as a support tool for healthcare professionals, potentially enhancing the quality of care and efficiency in healthcare systems. This perception can also empower patients, encouraging them to take an active role in their healthcare management [25]. On the other hand, these positive perceptions bear inherent risks, such as the potential for over-reliance on AI for critical health decisions, leading to neglect of professional medical advice. There’s also the risk of misinterpretation of information provided by ChatGPT, which could result in inappropriate self-diagnosis or treatment decisions. Moreover, trust in the technology implicitly extends to its handling of sensitive health data, raising significant privacy and security concerns [12, 26, 27]. Ethical implications, including the risk of algorithmic bias and the need for clear accountability, further complicate the integration of AI in healthcare decision-making. Thus, while the perceived competence and trustworthiness of ChatGPT in healthcare can be immensely beneficial, it also necessitates a cautious approach, balancing these advantages against the potential risks through continuous evaluation, adaptation, and the establishment of robust guidelines for safe and ethical use.

### Perception of users using ChatGPT for non-healthcare queries

For individuals who have not yet used ChatGPT for healthcare queries, the composite of factors including ’Trustworthy, Secure, Benefits outweigh risks, Satisfaction, Willing to take decisions, Intent to use, and Persuasive’ becomes particularly influential, reflecting a spectrum of expectations, perceived needs, and concerns about engaging with AI in a healthcare setting. Trustworthiness is paramount; potential users seek assurance that the AI system will reliably deliver accurate and relevant health information. Security is another critical factor, as concerns about the handling and protection of sensitive health data are heightened for those unfamiliar with the technology’s capabilities. The assessment of whether the benefits of using ChatGPT substantially outweigh any potential risks, such as misdiagnosis or privacy breaches, plays a crucial role in their decision-making process. Satisfaction, implying that the tool meets or exceeds user expectations in functionality and quality of output, is vital for those considering its adoption. The willingness to make informed health decisions based on the AI’s guidance reflects a user’s confidence in the technology’s ability to support critical health-related choices. Intent to use is indicative of a readiness to embrace new technology, influenced by how well users perceive the tool aligns with their health information needs. Lastly, persuasiveness is key; it’s not just about the tool providing information, but doing so in a compelling and convincing manner, crucial for building trust and acceptance in AI advice. For potential users, these factors collectively form a framework of assurance encompassing utility, risk management, and confidence in AI, underpinning their readiness to integrate such technology into their healthcare journey.

### User readiness for ChatGPT

The recent discovery of ChatGPT’s limitation [12] calls for urgent discussion about Technology Readiness Levels (TRLs) [28]. TRLs are a systematic metric used to assess the development stage of a technology, ranging from the conceptual stage (TRL 1) to full-scale deployment (TRL 9) [29]. In the traditional assessment of TRLs, the focus primarily lies on the technological development stages, from concept formulation to actual deployment. However, integrating user perspectives, as revealed in our study, into the TRL framework can offer a more holistic and practical approach to assessing technology maturity, especially for user-centric technologies like ChatGPT in healthcare applications. Technology readiness is not static; it evolves as the technology develops and as users’ needs and perceptions change. Incorporating user perspectives into TRL assessments allows for a dynamic and adaptive approach to evaluating technology readiness, ensuring that assessments remain relevant and reflective of both technological advancements and shifts in user attitudes. Our findings, highlighting the significance of factors such as ’Trustworthy’, ’Competent’, and ’Benefits outweigh risks’, underscore the necessity of aligning technology readiness with user readiness for the successful adoption of ChatGPT in healthcare settings. While the technological development of ChatGPT may be advanced, our analysis reveals that user perceptions, particularly trust and perceived utility, play a pivotal role in its acceptance and effective use. This highlights a crucial insight: technology readiness alone is insufficient. If the users are not ready, due to misconceptions or lack of awareness about the technology’s capabilities and utility, the full potential of ChatGPT may remain untapped, or worse, be misused. Therefore, our findings advocate for a balanced approach where both technological advancement and user readiness are harmonized. This involves not only enhancing the technical features of ChatGPT but also actively engaging with potential users to address their concerns, educate them about the technology’s capabilities, and integrate their feedback, thereby ensuring that the technology is not just technically sound but also aligns with user expectations and needs for successful implementation in healthcare contexts.

### Limitations

In enhancing and extending the discussion on the limitations of our study, it’s crucial to address both the methodological constraints and the statistical considerations of our approach. The discrepancy in sample sizes between the healthcare user group (n = 44) and the non-healthcare user group (n = 563) is a limitation. We advocate for a cautious interpretation of the results, particularly in applying our findings to a broader population of healthcare information seekers. Moreover, our sampling methodology, primarily through social media channels, might introduce a selection bias. The demographic that engages with healthcare-related AI through social media platforms may not accurately represent the broader population seeking healthcare information. Reliance on self-reported data is another methodological constraint. While self-reporting is a common and valuable tool in research, it inherently carries the risk of inaccuracies. Participants’ responses could be influenced by their perceptions, experiences, or even social desirability bias, which might affect the authenticity of the data. To mitigate some of these limitations, our study employed a Bayesian analytical approach. The Bayesian method is advantageous in dealing with smaller sample sizes [30]. Despite these methodological efforts, further research with a more diverse and larger sample, perhaps supplemented with objective usage metrics, would be beneficial to validate and expand our findings and to develop a more comprehensive understanding of how different user groups perceive and use AI in healthcare contexts.

## Conclusion

Our study findings suggest a clear demarcation in user expectations and requirements from AI systems based on the context of their use. In healthcare, where decisions have direct health implications, trustworthiness emerges as a paramount concern, emphasizing the need for AI systems that prioritize accuracy, clarity, and security. For non-healthcare contexts, while trustworthiness remains important, it encompasses a broader range of factors including efficiency, user experience, and novelty. This dichotomy provides valuable insights for AI developers and healthcare professionals. It suggests a tailored approach to AI system design and communication, one that is acutely sensitive to the context of use. Future research might focus on exploring these context-specific preferences in greater detail, potentially leading to more personalized and effective AI applications across various domains.

## Supporting information

S1 Checklist COREQ (COnsolidated criteria for REporting Qualitative research) checklist. (PDF)

S1 Dataset Minimal dataset. (CSV)
